# Supplementary material for: Iron Trace Elements Concentration in PM10 and Alzheimer’s Disease in Lima, Peru: Ecological Study
Source: Biomedicines. 2024 Sep 8;12(9):2043. doi: 10.3390/biomedicines12092043 (PMC11429173; doi:10.3390/biomedicines12092043)
Supplement: Supplementary file 1 [file biomedicines-12-02043-s001.zip › Table S1.pdf]

Table S1. Correlation analysis between Fe in PM_{10} with all the metals present in PM_{10} by district.

Trace element	Comas		Lima Downtown		Lince		El Agustino		SJM		Surco	
	Rho*	p-value	Rho*	p-value	Rho*	p-value	Rho*	p-value	Rho*	p-value	Rho*	p-value
**Be**	**0.370**	**<0.001**	-0.033	0.762	-0.012	0.906	-0.020	0.805	**0.579**	**<0.001**	-0.022	0.839
**Cd**	**0.351**	**<0.001**	0.108	0.324	-0.021	0.839	0.155	0.061	**0.368**	**<0.001**	-0.023	0.832
**Co**	**0.372**	**<0.001**	-0.032	0.768	-0.030	0.770	-0.005	0.995	**0.579**	**<0.001**	-0.014	0.897
**Mo**	0.077	0.465	-0.032	0.769	-0.063	0.541	-0.016	0.839	-0.038	0.667	-0.023	0.836
**Li**	**0.372**	**<0.001**	-0.032	0.769	-0.030	0.769	-0.004	0.995	**0.580**	**<0.001**	-0.023	0.836
**Ni**	**0.234**	**0.025**	**0.288**	**0.007**	-0.014	0.885	0.160	0.052	0.190	0.033	**-0.224**	**0.042**
**Sb**	0.201	0.055	0.037	0.736	-0.012	0.907	0.053	0.523	0.088	0.327	-0.023	0.835
**Se**	**0.372**	**<0.001**	-0.032	0.769	-0.030	0.769	-0.004	0.995	**0.580**	**<0.001**	-0.023	0.836
**Cr**	0.122	0.246	**0.232**	**0.032**	-0.053	0.603	**0.256**	**0.001**	0.157	0.079	0.058	0.600
**Cu**	**0.424**	**<0.001**	**0.584**	**<0.001**	**0.376**	**<0.001**	**0.187**	**0.012**	0.144	0.068	0.052	0.605
**Mn**	**0.479**	**<0.001**	0.077	0.427	0.159	0.079	**0.465**	**<0.001**	**0.221**	**0.005**	-0.060	0.546
**Pb**	**0.586**	**<0.001**	**0.530**	**<0.001**	**0.247**	**0.015**	**0.344**	**<0.001**	**0.386**	**<0.001**	-0.061	0.585
**Zn**	**0.494**	**<0.001**	**0.629**	**<0.001**	**0.546**	**<0.001**	**0.564**	**<0.001**	**0.208**	**0.016**	0.001	0.987

*Spearman correlation test.
Bold letters indicate significant correlation (p<0.05).
SJM: San Juan de Miraflores.
