# Supplementary figures and images for: Iron Trace Elements Concentration in PM10 and Alzheimer’s Disease in Lima, Peru: Ecological Study
Source: Biomedicines. 2024 Sep 8;12(9):2043. doi: 10.3390/biomedicines12092043 (PMC11429173; doi:10.3390/biomedicines12092043)

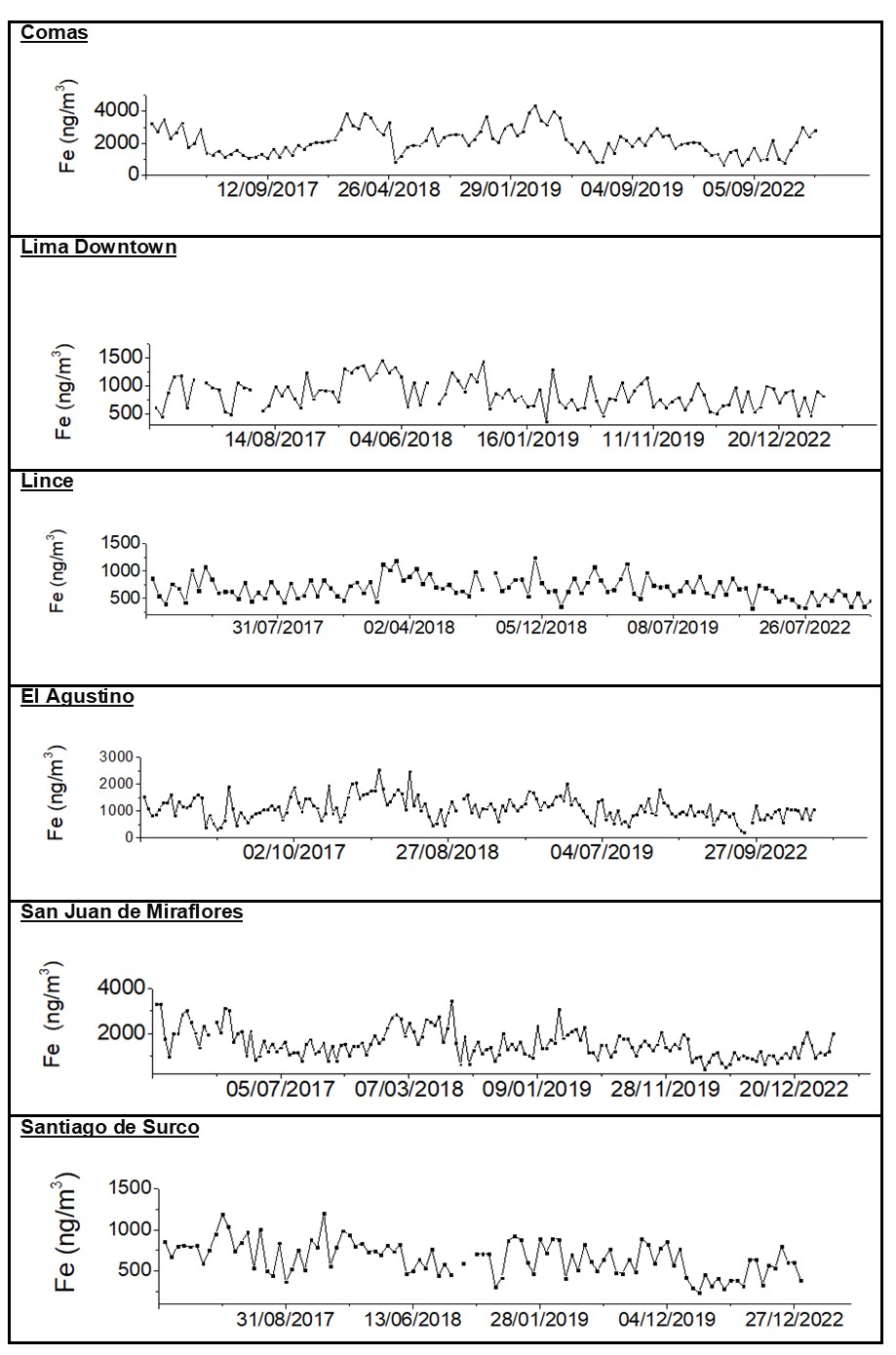

Supplement: Supplementary file 1 [file biomedicines-12-02043-s001.zip › Figure S1.jpg]

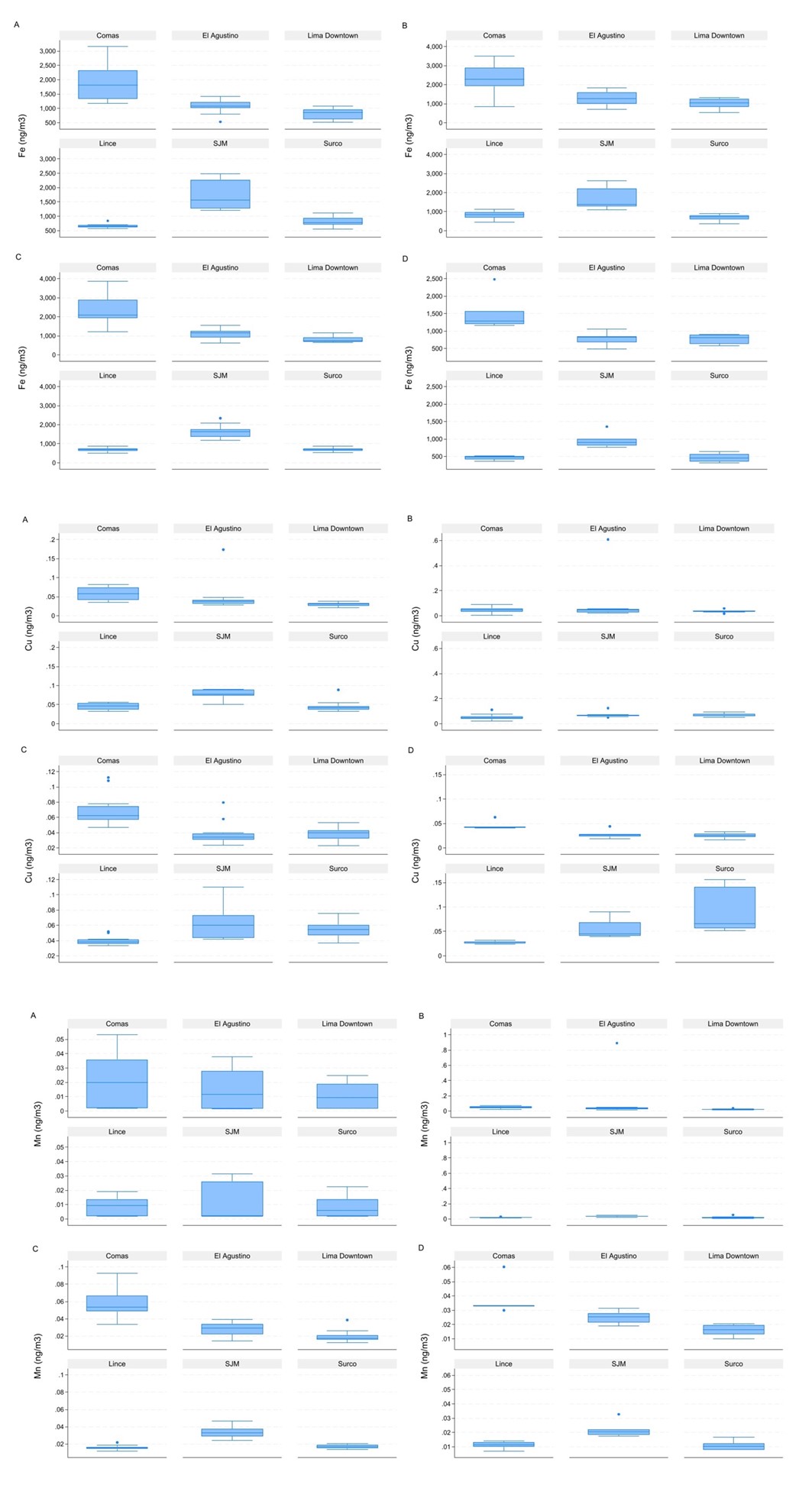

Supplement: Supplementary file 1 [file biomedicines-12-02043-s001.zip › Figure S2.jpg]
